# Lived experiences of type 2 diabetes self-management in Jordan: A qualitative study of sociocultural, religious, and healthcare influences

**DOI:** 10.1371/journal.pone.0350641

**Published:** 2026-06-05

**Authors:** Abdullah Hasan, Alanoud Alobaidly, Osama Alkouri, Fatma Refaat Ahmed

**Affiliations:** 1 College of Nursing, The Public Authority for Applied Education and Training, Kuwait, Kuwait; 2 Assistant Professor, College of Nursing and Health Sciences, Flinders University, Bedford Park, ‌‌Australia; 3 Associate Professor, Faculty of Nursing, Yarmouk University, Irbid,‌‌ Jordan; 4 College of Health Sciences, University of Sharjah, Sharjah, UAE; Mutah University, JORDAN

## Abstract

**Aim:**

To explore the experiences of Jordanian adults living with T2DM and understand the influence of sociocultural, religious, emotional, and healthcare factors on their daily self-management practices.

**Background:**

T2DM prevalence is alarmingly high and increasing in Jordan. However, there is a lack of understanding about the daily experiences and challenges faced by T2DM patients in the complex cultural and social environment. Understanding the experiences of T2DM patients in the complex cultural and social environment is crucial for the design and implementation of appropriate interventions.

**Methodology:**

A qualitative phenomenological approach was used for the study. Semi-structured in-depth interviews were conducted with 20 participants with T2DM. Participants were selected from the major tertiary care hospital in Northern Jordan. Reflexive thematic analysis, supported by NVivo software, was used for the data analysis. Triangulation, audit trail, peer debriefing, and member checking were used for the establishment of the trustworthiness of the study.

**Results:**

Six major themes were identified. Cultural pressures concerning food, challenges in adhering to the prescribed medication, religious and cultural influences, emotional and psychological burdens, gaps in healthcare services, and facilitators and coping strategies.

**Conclusion:**

Self-management practices for T2DM patients in Jordan are significantly influenced by cultural, religious, emotional, and healthcare factors. Interventions for T2DM patients in Jordan need to be appropriate and tailored to the culture and community.

**Implications for Nursing:**

It is the duty and responsibility of the nurse to provide appropriate education and advice based on the culture and community. Family involvement in the care process for T2DM patients in Jordan is crucial.

## Introduction

Type 2 diabetes mellitus (T2DM) is a growing public health challenge worldwide, with 537 million adults affected in 2021, projected to rise to 783 million by 2045 [1]. In the Middle East, Jordan faces one of the highest burdens of diabetes, with the International Diabetes Federation (IDF) estimating that 20.5% of Jordanian adults aged 20–79, over 1.08 million people, will be living with diabetes in 2024 [2]. Non-communicable diseases (NCDs), including diabetes, account for approximately 78% of all deaths in Jordan, with diabetes alone contributing around 7% of mortality [3,4]. Nevertheless, although epidemiological statistics may be informative in terms of the magnitude of the issue, they do not shed much light on the lives that individuals lead living with diabetes.The increasing prevalence of T2DM in Jordan is closely associated with lifestyle changes, urbanization, and dietary patterns that favor high-calorie, nutrient-poor foods [5]. Obesity and physical inactivity are widespread, creating significant challenges for effective disease control [6]. These factors not only heighten risk but also complicate daily self-management, which is essential for preventing complications and maintaining quality of life.Self-management of T2DM encompasses several interconnected behaviors: following dietary recommendations, adhering to medications or insulin regimens, engaging in physical activity, and regularly monitoring blood glucose [7,8]. In Jordan, however, these practices are shaped by broader cultural and social contexts. Family roles in health decision-making, stigma associated with chronic illness, and communal eating practices may make it difficult to follow dietary or medication plans consistently [9,10]. Religious obligations such as fasting during Ramadan also require careful adjustments to diet, medication schedules, and glucose monitoring, which many patients find challenging [11].

Self-management of T2DM entails active participation in a number of health-related activities such as diet control, medication or insulin administration, physical exercises, and blood sugar monitoring (McCallion, 2022; Nguyen, 2025). In many cases, the current literature has viewed these behaviors from an individualistic point of view whereby they are affected by knowledge, motivation, or adherence. Despite this, it is possible that a sociocultural approach to health behavior may better reflect the conditions that affect the behavior of people living in collectivist and familistic societies. The sociocultural model of health behavior [12]posits that a person’s health-related behaviours are influenced not only by their knowledge but also by familial expectations, societal norms, religion, gender, economy, and healthcare systems.This conceptual lens is particularly relevant in Jordan and similar Arab contexts, where food, family, hospitality, religion, and community life strongly influence daily routines. Communal eating practices, traditional high-calorie meals, and expectations to accept food during family gatherings may create tension between dietary advice and social belonging (Abuhammour, 2024; Rais, 2024). Similarly, religious practices such as Ramadan fasting may require adjustment of medication, diet, and glucose monitoring, yet patients’ decisions are often influenced by faith commitments, family expectations, and fear of social judgment (Arslan & Aydın, 2024). Gender norms, limited access to safe environments for physical activity, stigma surrounding insulin use, and inconsistent follow-up within healthcare services may further complicate self-management. These factors indicate that diabetes care cannot be fully understood without considering the social and cultural environment in which patients make daily decisions.

While quantitative studies describe the prevalence and risk factors of diabetes in Jordan, there is limited qualitative research exploring patients’ lived experiences of daily self-management. Collectively, all these studies highlight that diabetes management is dependent on diet, lack of exercise, obesity, compliance, health literacy, and access to health care (Al-Awwad et al., 2021; Al-Azayzih et al., 2023; Al-Qerem et al., 2024; Al-Sahouri et al., 2019). However, this literature remains fragmented. Little is known about how individuals with T2DM perceive the barriers and facilitators to diet control, adherence to medication, maintaining physical activity, and monitoring glucose within their sociocultural environment. Furthermore, patients’ views on what motivates and sustains self-management, and what they consider most important in managing their condition, remain underexplored. This study uses a phenomenological approach to understand the real-life experiences of Jordanian adults with type 2 diabetes, highlighting how they manage the daily challenges of self-management including diet, medication, physical activity, and glucose monitoring—within their unique sociocultural and religious context. By exploring the personal, psychological, cultural, and social factors that shape patients’ practices, as well as the barriers and facilitators they encounter, the research aims to illuminate what matters most to individuals in managing their diabetes. The insights gained are intended to guide culturally sensitive, patient-centered interventions that empower patients to take control of their health and improve diabetes care in Jordan.

## Methods

### Study design

This study employed a qualitative phenomenological design to explore the lived experiences of Jordanian adults with T2DM. This study was informed by an interpretive (hermeneutic) phenomenological approach, which seeks to understand how individuals make sense of their lived experiences within their sociocultural context. The methodological approach was grounded in the phenomenological tradition as outlined by Maltby, Williams [13], Tanwir, Moideen [14], emphasizing individuals’ subjective understandings of their daily self-management practices, challenges, facilitators, and sociocultural influences shaping these experiences.

### Study setting

The study was conducted in outpatient diabetes clinics in the largest tertiary hospital in the North of Jordan. This institution was selected based on its established reputation for providing diabetes care and their capacity to represent diverse healthcare contexts and patient populations. A total sample of 20 participants were included into the study.

The selected outpatient clinics served high volumes of patients with T2DM and were well positioned for recruitment due to their focus on chronic disease management. All interviews were conducted in private consultation rooms within these clinics to ensure confidentiality, minimize interruptions, and create a comfortable environment conducive to open and detailed discussion.

### Study participants

Participants were recruited using purposive sampling to include Jordanian adults (≥18 years) with a confirmed diagnosis of T2DM. The sample was designed to capture diversity in educational attainment, socioeconomic status, geographic residence (urban vs. rural), and duration of diabetes (newly diagnosed to long-term management). Eligible participants were medically stable and able to communicate in Arabic.

Exclusion criteria comprised individuals with severe or terminal comorbidities (e.g., advanced cancer, end-stage renal disease), active psychiatric illness, or a history of organ transplantation to ensure focus on diabetes-related experiences in order to concentrate on the self-management of diabetes, and not the self-management of complicated comorbid conditions.

A total of 20 participants were ultimately recruited, consistent with qualitative phenomenological standards [15], allowing sufficient depth for thematic saturation (refers to the stage where any further interview does not reveal new themes or subthemes because all the possible themes and subthemes have already been identified through previous interviews). Recruitment continued until no new themes emerged, ensuring comprehensive representation of the sociocultural, emotional, and systemic dimensions of diabetes self-management in Jordan.

### Data collection

Data were collected through semi-structured, in-depth individual interviews designed to elicit both personal and collective dimensions of self-management. Each interview lasted approximately 45–60 minutes and explored topics such as coping strategies, stigma, health literacy, and the influence of cultural or religious values on disease management.

Interviews were conducted in Arabic or English, depending on participant preference, and were audio-recorded with written informed consent. Arabic interviews were transcribed verbatim and, where necessary, translated into English for analysis. To ensure linguistic accuracy and preserve meaning, translations were reviewed by bilingual researchers familiar with the cultural context.

To enhance comfort and cultural sensitivity, interviews were conducted by a gender-concordant researcher whenever possible. The primary researcher also maintained detailed field notes to document nonverbal cues and contextual observations.

The interview guide was developed following a comprehensive review of relevant literature and consultations with Jordanian healthcare professionals to ensure cultural appropriateness. The guide explored core themes related to daily routines, dietary habits, medication and insulin adherence, physical activity, and glucose monitoring. Broader contextual influences—such as family roles, religious practices (including Ramadan fasting), and healthcare interactions—were also addressed. The semi-structured interview guide used for data collection is provided in S1 Text.

The primary researcher completed training in culturally sensitive and trauma-informed interviewing. When applicable, all interviews adhered to public health safety protocols (e.g., physical distancing, use of personal protective equipment) following the Jordanian Ministry of Health’s COVID-19 guidelines.

Data saturation was assessed iteratively during data collection and analysis, with no new codes or themes emerging after approximately the 17th interview, indicating sufficient depth and breadth of the data.

### Recruitment procedure

Recruitment was facilitated through collaboration with clinic physicians and administrators. Physicians identified eligible patients through the clinic’s electronic medical records, and the clinic nurse approached them during routine appointments to introduce the study and distribute the Participant Information Sheet. A total of 24 eligible patients were approached to participate in the study. Of these, 20 agreed to participate and completed the interviews, while 4 declined due to time constraints or lack of interest.

Interested patients were encouraged to discuss participation with family members before contacting the researcher to schedule an interview.

At the interview session, the researcher explained the study objectives, answered questions, and obtained written informed consent. Participants also completed a short demographic questionnaire that captured age, gender, education, place of residence, and duration of diabetes.

### Data analysis

Interview data were analyzed using semantic and inductive thematic analysis as proposed by [16,17]. This analytic framework was selected for its capacity to capture complex, patterned meanings within qualitative data and to illuminate both shared and individual experiences of diabetes self-management.

Analysis proceeded through six iterative phases. First, transcripts were transcribed verbatim, and the researcher repeatedly read and listened to recordings to achieve immersion and familiarity. Next, initial codes were generated inductively from the data to capture significant features of participants’ narratives, including beliefs, coping strategies, and contextual influences.

Reflexive thematic analysis, as outlined by Braun and Clarke, was used to identify patterns of meaning across participants’ narratives. This approach aligns with interpretive phenomenology, as it supports an in-depth exploration of subjective experiences while recognizing the researcher’s active role in meaning-making. It enabled the development of themes that capture both shared and individual aspects of living with type 2 diabetes within a sociocultural context.

Codes were then collated into candidate themes that reflected patterned responses across participants. Themes were subsequently reviewed and refined to ensure coherence and representativeness, followed by the definition and naming of each theme to articulate its contribution to understanding the lived experience of T2DM. The final phase involved constructing a rich interpretive narrative supported by illustrative quotations, contextualized within Jordan’s cultural and healthcare environment.

NVivo software (QSR International) was employed to manage and organize data systematically. To enhance analytic rigor, a second qualitative researcher independently coded a subset of transcripts. Coding discrepancies were resolved through discussion and consensus.

### Trustworthiness and rigor

The study’s rigor was guided by Alexander [18, 19] criteria of credibility, dependability, confirmability, and transferability. Credibility was strengthened through prolonged engagement, member checking, and methodological triangulation. The researcher established rapport with participants, enabling open discussion, and conducted member checks by summarizing key points during interviews for participant validation. Triangulation of diverse participant backgrounds enhanced representativeness.

Dependability was ensured by maintaining an explicit audit trail detailing the research process, including methodological decisions, coding procedures, and analytic reflections. Peer debriefing sessions with experienced qualitative researchers provided external scrutiny of analytic decisions.

Confirmability was enhanced through reflexive journaling, documenting the researcher’s assumptions and reactions throughout data collection and analysis. An independent qualitative expert audited selected transcripts and coding frameworks to ensure interpretations were grounded in participants’ accounts.

Transferability was supported by providing thick descriptions of participants’ contexts, including sociocultural and religious factors influencing diabetes management in Jordan. These detailed contextual insights enable readers to assess applicability to other Middle Eastern or Muslim-majority populations.

### Reflexivity statement

Members of the research team were experienced clinicians and academics with backgrounds in nursing and diabetes-related care. This expertise supported a deeper understanding of participants’ experiences; however, it also required careful reflexivity to avoid preconceptions influencing the analysis. The primary researcher maintained a reflexive stance throughout the study, acknowledging potential assumptions and their influence on data interpretation. Reflexive journaling was used to document personal perspectives and analytic decisions. Reflexivity was further supported through maintaining reflexive notes, engaging in regular peer debriefing on evolving interpretations, and ensuring that findings remained grounded in participants’ accounts. We recognized the potential influence of these perspectives on the applicability of our findings, which are most relevant to adults with T2DM in a medically stable condition.

### Ethical considerations

This study was conducted in accordance with ethical standards and was approved by the Institutional Review Board (IRB) of King Abdullah University Hospital (Approval ID: Oct2025/186–32; approved on 1 October 2025).

All participants provided informed consent prior to participation, including consent for the use and publication of anonymized interview excerpts, themes, and codes for research and academic purposes. All quotations were fully de-identified before analysis and reporting, and no names, initials, nicknames, exact ages, dates, addresses, contact details, institutional identifiers, ID numbers, or other potentially identifying information are included in the manuscript. Participant quotations are presented using non-identifiable codes only, in accordance with ethical standards and confidentiality requirements.. Participants were informed of their right to withdraw at any time without penalty.

Confidentiality was maintained through anonymization of all transcripts and the use of coded identifiers. Digital data were stored on encrypted, password-protected devices accessible only to the research team, while hard copies were secured in locked cabinets. Personally, identifying information was stored separately from study data.

Data will be retained for five years following study completion in accordance with Jordanian data protection regulations, after which digital files will be permanently deleted, and hard copies destroyed. In the event of emotional distress during interviews, participants were offered the option to pause or discontinue and were referred to appropriate mental health services. No financial compensation was provided to avoid coercion; participation was entirely voluntary and motivated by the potential contribution to improving diabetes care in Jordan.

## Results

The qualitative analysis yielded six overarching themes and seventeen subthemes that collectively capture the complexity of diabetes self-management within participants’ sociocultural context (Table 1). These themes move beyond descriptive accounts to reflect the deeper interplay between individual behaviors and the cultural, religious, emotional, and healthcare environments in which they are embedded. Together, they illustrate how self-management is shaped by dynamic and interconnected influences, ranging from cultural expectations and treatment-related challenges to emotional burden, healthcare system limitations, and available coping resources.

**Table 1:** Summary of Themes, Subthemes, and Illustrative Quotes.

Theme	Subtheme	Quotes
**Theme 1: Cultural Pressure Around Food**	**Subtheme 1.1: Social and Cultural Expectations**	“I know I should avoid sweets, but at family gatherings food is everywhere. I feel bad saying no” (P3).“When someone offers mansaf, it’s hard to refuse. People might think I’m rude” (P7).“The problem is the sweets and food at our gatherings, like konafa and mansaf. It feels like we’re expected to eat, and it’s hard to resist” (P11).
	**Subtheme 1.2: Barvriers to Physical Activity**	“I want to walk daily, but I’m tired after work and there’s no safe place nearby” (P4). “For women, walking alone outside isn’t always acceptable in our traditions” (P9).“The gym is too far and too expensive” (P16).“I’m always working, and when I get home, I’m exhausted and have no time or energy for anything else” (P18).
**Theme 2: Medication Adherence and Treatment Barriers**	**Subtheme 2.1: Forgetfulness and Treatment Fatigue**	“After years of pills, I get tired of taking them every day” (P1).“Life is busy with stress and responsibilities. Sometimes I take it in the morning, sometimes in the evening, and sometimes I just don’t take it at all” (P13).“At the beginning, I took it regularly, but now I forget and only take it when my symptoms get really bad” (P22).
	**Subtheme 2.2: Stigma and Fear Around Insulin**	“I was scared to start insulin because people say it means your diabetes is very bad” (P5).“I avoid injections in public because I don’t want people to see me as sick” (P17).
	**Subtheme 2.3: Self-Adjustment of Medications**	“If I feel weak, I change the dose myself without asking the doctor” (P12).“I reduce doses to avoid side effects or increase them when sugar is high” (P19).
**Theme 3: Religious and Cultural Influences**	**Subtheme 3.1: Fasting During Ramadan**	“Even though my doctor said not to fast, I still do. It’s part of my faith” (P6).“My family expects me to fast, so I cannot refuse” (P10).“If people see me not fasting, they will expose me and laugh. It’s impossible for me to break the fast” (P15).“Where is my manhood if I break the fast in Ramadan?” (P20).“It’s haram to break the fast, and God will take care of my health” (P23).
	**Subtheme 3.2: Faith and Trust in God**	“I leave my health to God; He knows best” (P2).“I pray and trust God to take care of me” (P8).“The sickness won’t get me. Only God decides when we die” (P14).
**Theme 4: Emotional and Psychological Burden**	**Subtheme 4.1: Anxiety and Guilt**	“Living with diabetes makes me worry all the time” (P21).“When my blood sugar goes high, I feel stressed and worry about losing my leg” (P24).“I feel guilty after eating traditional sweets” (P7).
	**Subtheme 4.2: Social Exclusion**	“I feel left out when I can’t eat like my family” (P3).“My relatives think I exaggerate when I say I must follow a diet” (P9).
	**Subtheme 4.3: Stress and Blood Sugar**	“When I’m stressed, my sugar rises. It feels like a cycle I can’t stop” (P11).“When my sons fight, I get upset and my sugar rises” (P18).
**Theme 5: Gaps in Healthcare Support**	**Subtheme 5.1: Rushed Consultations**	“Doctors are always in a hurry. They just say my sugar is high and give me pills” (P4).“There are thirty people waiting; the doctor sees me for five minutes, then I just get my medicine and leave” (P13).
	**Subtheme 5.2: Lack of Culturally Tailored Advice**	“Nobody taught me how to manage during Ramadan. I had to ask friends” (P10).“Most advice doesn’t fit our lifestyle” (P15).
	**Subtheme 5.3: Limited Follow-up**	“I only see the doctor every few months. No one checks on me in between” (P16).“I don’t have anyone to take me to the doctor; it’s hard for me to go by myself” (P19).“I only go to the doctor when I get a wound on my leg or problems with my eyes” (P22).
**Theme 6: Facilitators and Coping Strategies**	**Subtheme 6.1: Family Support**	“My son takes me walking in the evenings” (P5).“My wife cooks lighter meals so I can join the family” (P9).“My husband checks my sugar every morning so I don’t forget” (P12).
	**Subtheme 6.2: Religious Faith**	“Praying and reading the Quran calms me when I’m stressed” (P8).“I tell myself this is a test from God, so I have to stay strong” (P14).
	**Subtheme 6.3: Personal Coping Strategies**	“I check my sugar before meals to know what I can eat” (P11).“I set alarms so I don’t forget my meds” (P20).

### Theme 1: Cultural pressure around food

This theme highlights how sociocultural norms and everyday environments shaped participants’ ability to follow lifestyle recommendations. Food and social practices were deeply tied to identity, hospitality, and belonging, creating tension between cultural expectations and medical advice. Physical activity was also constrained by social norms, limited infrastructure, and competing demands, reflecting how health behaviors were embedded within broader cultural and structural contexts. Significantly, the theme was closely associated with the limitations of the healthcare system, in that the challenges that the participants experienced with regard to their eating and physical activity habits were aggravated by the impersonal nature of the recommendations received from the clinic.

### Subtheme 1.1: Social and cultural expectations

Participants described strong cultural expectations around communal eating that made dietary adherence challenging. Traditional foods and social gatherings were central to family life, creating pressure to eat even when aware of health risks. As one participant said, “I know I should avoid sweets, but at family gatherings food is everywhere. I feel bad saying no” (P3). Another added, “When someone offers mansaf, it’s hard to refuse. People might think I’m rude” (P7).

Many felt compelled to conform, particularly during celebrations: “The problem is the sweets and food at our gatherings, like konafa and mansaf. It feels like we’re expected to eat, and it’s hard to resist” (P11). These cultural and social norms often outweighed individual health intentions. The stress experienced by the subjects also translated into emotional strain as the subjects felt guilty, frustrated, and socially isolated during their attempts to adhere to the dietary advice. Therefore, the study demonstrates that failure to adhere to dietary guidelines was not only due to a lack of knowledge and motivation but also to a combination of emotional and cultural factors.

### Subtheme 1.2: Barriers to physical activity

Physical activity was constrained by fatigue, safety concerns, and limited facilities. One participant noted, “I want to walk daily, but I’m tired after work and there’s no safe place nearby” (P4). Gender norms also restricted women’s opportunities: “For women, walking alone outside isn’t always acceptable in our traditions” (P9).

Financial and logistical factors compounded these challenges: “The gym is too far and too expensive” (P16). Time pressures and exhaustion from work and family responsibilities further hindered participation: “I’m always working, and when I get home, I’m exhausted and have no time or energy for anything else” (P18).

Together, these factors positioned physical activity as a low priority amid competing cultural and socioeconomic demands. In essence, the barriers were interconnected with the constraints of the healthcare system since the participants were told to do exercise despite not being offered any viable alternatives. This was so because no consideration was taken for their safety, cultural constraints, financial capability, energy level, or suitable place to exercise.

### Theme 2: Medication adherence and treatment barriers

This theme indicates that adherence to diabetes treatment was challenged by both individual and social factors. Forgetfulness, emotional fatigue, stigma, and self-directed medication adjustments reflected the complexity of sustaining long-term treatment in the context of daily stress, limited support, and health system gaps. The use, delay, or adjustment of treatment was thus affected by the very same wider environment described in other themes, namely social stigma, emotional fatigue, religion, and lack of follow-up.

### Subtheme 2.1: Forgetfulness and treatment fatigue

Participants reported declining adherence over time due to treatment fatigue and competing daily demands. “After years of pills, I get tired of taking them every day” (P1), one participant said. Others skipped doses when feeling well or overwhelmed: “Life is busy with stress and responsibilities. Sometimes I take it in the morning, sometimes in the evening, and sometimes I just don’t take it at all” (P13).

Medication was often taken only when symptoms became severe: “At the beginning, I took it regularly, but now I forget and only take it when my symptoms get really bad” (P22). These accounts highlight how long-term management fatigue and lifestyle pressures undermine consistent adherence.

### Subtheme 2.2: Stigma and fear around insulin

Fear and stigma surrounded insulin use, with several participants viewing it as a sign of disease severity or weakness. One explained, “I was scared to start insulin because people say it means your diabetes is very bad” (P5). Others avoided injections in public due to embarrassment or self-consciousness about appearing “sick” (P17).

### Subtheme 2.3: Self-Adjustment of Medications

Participants frequently modified medication doses without medical advice, relying on personal judgment or symptoms. “If I feel weak, I change the dose myself without asking the doctor” (P12). Others reduced doses to avoid side effects or increased them to manage high blood sugar (P19). Such practices reflected attempts at self-control but posed potential safety risks in the absence of professional guidance.

### Theme 3: Religious and cultural influences

This theme shows how faith influences health choices and coping. Religious practices like fasting sometimes conflict with medical advice, while trust in God offers comfort and helps people cope emotionally. Religious influence did not exist independent of other themes but was interwoven with family expectations, health care communication, emotional coping, and treatment choices, especially during Ramadan. Religious influence did not exist independent of other themes but was interwoven with family expectations, health care communication, emotional coping, and treatment choices, especially during Ramadan.

### Subtheme 3.1: Fasting during Ramadan

Religious commitment strongly influenced fasting behaviors, often overriding medical advice. “Even though my doctor said not to fast, I still do. It’s part of my faith” (P6). Social and familial expectations reinforced this behavior: “My family expects me to fast, so I cannot refuse” (P10).

Fear of social judgment also played a role: “If people see me not fasting, they will expose me and laugh. It’s impossible for me to break the fast” (P15). For some men, fasting was tied to identity and masculinity: “Where is my manhood if I break the fast in Ramadan?” (P20).

Faith and divine trust guided many decisions: “It’s haram to break the fast, and God will take care of my health” (P23). These findings underscore the powerful role of faith and community in shaping health behavior.

### Subtheme 3.2: Faith and Trust in God

Faith functioned as a coping mechanism, providing emotional reassurance and acceptance. Participants often entrusted their health to God: “I leave my health to God; He knows best” (P2). Prayer and spiritual reflection helped reduce anxiety: “I pray and trust God to take care of me” (P8).

This spiritual surrender fostered resilience and optimism: “The sickness won’t get me. Only God decides when we die” (P14).

### Theme 4: Emotional and psychological burden

This theme highlights the emotional impact of diabetes, including anxiety, guilt, and feeling isolated. Stress and cultural food pressures affect both mental health and blood sugar, making people feel less in control. The emotional strain seemed to cut across all the other themes, including culture pressure about eating, treatment fatigue, religious tension, and insufficient health care support.

### Subtheme 4.1: Anxiety and guilt

Living with diabetes generated persistent worry and guilt, particularly around diet and blood sugar control. “Living with diabetes makes me worry all the time” (P21). Fear of complications intensified distress: “When my blood sugar goes high, I feel stressed and worry about losing my leg” (P24).

Many described guilt after consuming traditional sweets, reflecting the emotional toll of balancing health with cultural food practices (P7).

### Subtheme 4.2: Social exclusion

Dietary restrictions often led to social isolation. Participants avoided gatherings or felt misunderstood by others: “I feel left out when I can’t eat like my family” (P3), and “My relatives think I exaggerate when I say I must follow a diet” (P9). This lack of social inclusion deepened emotional strain. This relates to the pressure that exists culturally about food and stresses that self-management might pose a challenge to one’s sense of belonging and inclusion in the community and the family. It was noted that without any family or culturally-oriented counselling, the participants had to deal with this conflict on their own.

### Subtheme 4.3: Stress and Blood Sugar

Participants perceived a direct relationship between emotional stress and glucose fluctuations: “When I’m stressed, my sugar rises. It feels like a cycle I can’t stop” (P11). Family conflicts and daily stressors contributed to this cycle: “When my sons fight, I get upset and my sugar rises” (P18), emphasizing the need for emotional support in diabetes care.

### Theme 5: Gaps in Healthcare Support

This theme highlights participants’ dissatisfaction with healthcare interactions, characterized by brief consultations, limited follow-up, and culturally insensitive advice. These experiences indicate systemic barriers that hinder patient engagement, self-efficacy, and culturally appropriate diabetes care.

### Subtheme 5.1: Rushed Consultations

Participants described brief, impersonal consultations that limited understanding and engagement. “Doctors are always in a hurry. They just say my sugar is high and give me pills” (P4). Overcrowded clinics further constrained interactions: “There are thirty people waiting; the doctor sees me for five minutes, then I just get my medicine and leave” (P13).

### Subtheme 5.2: Lack of Culturally Tailored Advice

Advice was often perceived as generic and culturally mismatched. “Nobody taught me how to manage during Ramadan. I had to ask friends” (P10). Others felt recommendations ignored local foods and customs: “Most advice doesn’t fit our lifestyle” (P15).

### Subtheme 5.3: Limited Follow-up

Follow-up care was inconsistent, leaving patients largely self-reliant. “I only see the doctor every few months. No one checks on me in between” (P16). Barriers such as transport and family responsibilities further limited access: “I don’t have anyone to take me to the doctor; it’s hard for me to go by myself” (P19).

Some sought care only when complications arose: “I only go to the doctor when I get a wound on my leg or problems with my eyes” (P22). The lack of follow-up led to medication adjustment, seeking advice from unofficial sources, late care-seeking, and low self-confidence. This illustrates that the problems within the healthcare system were not just present alongside personal barriers; rather, they played a role in how individuals coped with their challenges.

### Theme 6: Facilitators and Coping Strategies

This theme reflects the resources that supported participants’ ability to manage diabetes. Family encouragement, religious faith, and personal initiative emerged as key facilitators, illustrating how social and spiritual networks fostered motivation, resilience, and self-efficacy in everyday management.

### Subtheme 6.1: Family Support

Family involvement was a major facilitator of self-management. Relatives reminded participants to take medication, encouraged physical activity, and prepared healthier meals. “My son takes me walking in the evenings” (P5), and “My wife cooks lighter meals so I can join the family” (P9).

Such support provided both motivation and accountability, reinforcing adherence: “My husband checks my sugar every morning so I don’t forget” (P12). In contrast to the previous finding in which family and social functions can be seen to generate pressure to consume unhealthy foods or fast even against medical advice, here, the role played by family is that of both an inhibitor and a facilitator of healthy eating behaviors.

### Subtheme 6.2: Religious Faith

Faith was a critical emotional resource. Prayer and trust in God fostered calm and perseverance: “Praying and reading the Quran calms me when I’m stressed” (P8). Many interpreted illness as a divine test requiring patience and strength: “I tell myself this is a test from God, so I have to stay strong” (P14).

### Subtheme 6.3: Personal coping strategies

Participants described proactive self-management behaviors, including monitoring glucose, portion control, and scheduling reminders. “I check my sugar before meals to know what I can eat” (P11), and “I set alarms so I don’t forget my meds” (P20).

These individualized routines reflected agency and adaptability in managing diabetes within everyday constraints.

## Discussion

This study revealed how sociocultural factors, religious beliefs, emotional factors, and limitations within the healthcare system influence diabetes self-management among adults in Jordan. Rather than being understood as an isolated medical condition, diabetes appears to be experienced as a challenge embedded within social and cultural contexts. Viewed through a socio-ecological perspective, these findings suggest that diabetes self-management is shaped not by knowledge or motivation alone, but by interacting with influences at multiple levels. At the interpersonal level, family dynamics—such as shared meals, expectations to accept food, and encouragement or pressure from relatives—strongly influenced dietary practices, fasting, and medication routines. At the organizational level, brief consultations and generic advice limited opportunities for personalized support. At the community level, unsafe environments, financial constraints, and gender norms further restricted physical activity. Together, these findings highlight that self-management is embedded within a broader social and structural context, consistent with ecological and multilevel models of care. Recent evidence supports this interpretation, showing that diabetes self-management is shaped by interacting psychological, social, and physiological factors rather than individual behaviour alone [20], that social determinants such as environment and socioeconomic conditions significantly influence self-care behaviors [21,22], and that interventions leveraging multilevel social resources lead to improved self-management outcomes [22,23].

The participants’ accounts also showed a clear tension between recommendations for healthy living and the realities of Arab social structures, including family expectations, social meals, religious practices, and resource constraints. This created a complex environment in which diabetes self-management had to be negotiated on a daily basis.

Theme 1 showed that cultural pressure around food remains a major barrier to lifestyle modification, especially during gatherings where hospitality is expressed through abundant traditional dishes such as mansaf and festive sweets. These findings were also reported in recent studies conducted in Jordan and the region [24,25]. Participants reported that social pressures were often stronger than medical recommendations when it came to food choices. Overall, the studies suggest that the motivation to adhere to a particular diet is not only an individual choice but is also connected to social identity. For some people, refusing food at social gatherings is considered rude and may create emotional distress and guilt. Therefore, for behaviour change to be effective, it is important that it is culturally appropriate and that it targets not only the individual but also the family and the community. Community-based interventions that promote the idea that traditional foods can be healthy may be more effective than conventional approaches. Notably, barriers to physical activity were identified as gender-related, fatigue-related, and safety-related. This is in line with previous findings among women in Jordan and other Arab countries [26,27]. For example, women have identified social restrictions and a lack of safe walking environments as barriers to physical activity [28]. As a result, physical activity is often not prioritized in comparison with work and family obligations.

Theme 2 describes challenges in adherence to medication regimens, including forgetfulness, fatigue, and self-initiated modifications to treatment. These behaviors have been widely studied and documented in Jordan and the Middle East [29,30]. Stigma associated with insulin therapy has also been recognized [31,32]. In this context, fear of insulin initiation is often perceived as a sign that the illness is severe or has “failed” to respond to treatment. Evidence of this has been reported in studies from Saudi Arabia, Bahrain, and Qatar [33,34]. Improvements in continuity of care, including greater involvement of nurses in diabetes management, may also reduce the need for dose adjustments.

Theme 3 reflects the influence of religious beliefs. Participants reported continuing to fast during Ramadan despite medical advice against it, owing to religious beliefs, familial pressures, social expectations, and fear of stigmatization. These findings are consistent with studies from the Middle East, which suggest that fasting is influenced more by social and religious factors than by medical advice alone [35,36]. At the same time, faith acted as a coping mechanism by providing emotional support and strength. This dual role of faith makes it important to consider its influence in patient education. Joint efforts with religious scholars and community leaders—an approach advocated in regional Ramadan–diabetes guidance—could improve the safety of fasting practices and strengthen patients’ engagement with healthcare providers [37].

Theme 4 illustrated the emotional and psychological burden of diabetes. Participants’ experiences of anxiety, guilt, and exclusion related to their dietary habits were consistent with regional literature, which reports high levels of diabetes distress caused by fear of complications, pressure to comply with social norms, and limited emotional support from family members and the community [38]. Participants’ observations of the effects of stress on blood glucose levels were also consistent with the biomedical literature [39], which reports the impact of psychological stress on blood glucose levels. These findings highlight the importance of addressing the psychological dimensions of diabetes management, as well as the contribution of the family to disease management, especially when the family’s cultural background strongly shapes members’ emotional states.

Theme 5 revealed gaps in healthcare support, including rushed consultations, generic advice, and insufficient follow-up, all of which contributed to patients feeling marginalized within the healthcare system. These challenges are consistent with the current study in Jordan, which also reported overburdened clinics, reduced physician-patient interaction time, and advice that was not aligned with cultural realities. The lack of access to continuous follow-up care also reinforced patients’ tendency to self-manage without professional support. Improving primary healthcare infrastructure, including continuity models involving nurses, has the potential to mitigate these challenges [40–42].

Theme 6 refers to the contribution of facilitators and coping factors, including family support, faith, and individual initiative. These are similar to the factors identified in Arab communities, where the family plays an important role in managing medication, meals, and daily routines [43,44]. Therefore, working with families to strengthen their role in these areas may improve outcomes. The proactive behaviours demonstrated by participants, such as using medication reminders and monitoring blood glucose levels before meals, could be supported through digital health technologies. Similar findings have been reported in previous studies [42,44–46].

Recent qualitative evidence from Mexico supports this interpretation by highlighting that, for people living with type 2 diabetes, self-management is experienced not only as a clinical responsibility but also as a deeply personal and often demanding part of everyday life. Participants in that study described how continuous glucose monitoring helped them better understand their blood glucose patterns, feel more in control, and enhance their sense of safety. At the same time, they also spoke about the challenges they faced, including fatigue, emotional strain, and difficulties related to the cost and use of technology. These findings underscore that patient-centered care needs to go beyond glycemic control to also acknowledge and address the day-to-day practical and emotional realities of living with a chronic condition [47].

This study adds to existing regional literature by showing that the barriers to diabetes self-management in Jordan are not isolated problems but interconnected social, religious, emotional, and health-system pressures that shape daily decision-making. Previous regional studies have documented physical activity barriers, diabetes distress, and concerns about patient-centered care, but this study integrates these issues into one culturally grounded account of lived experience, demonstrating how food practices, Ramadan fasting, stigma, fatigue, and limited follow-up interact in everyday self-management. Recent regional evidence similarly indicates that diabetes self-management is influenced by sociocultural and environmental factors, and that patient-centered, culturally responsive care is associated with better patient experience and satisfaction [38,41,48,49].

From a clinical perspective, these findings highlight the importance of delivering diabetes education in a way that meaningfully engages both patients and their families, given the central role of family practices and support in shaping everyday self-management. Healthcare professionals should aim to provide guidance that is grounded in patients’ lived realities, including culturally sensitive advice on traditional dietary practices and Ramadan fasting, while also paying closer attention to diabetes-related distress, medication adherence, and opportunities for physical activity. The findings also underscore the importance of delivering culturally responsive, patient-centred diabetes care. Nurses should actively involve family members in education and care planning, acknowledging their key role in supporting self-management. Dietary counselling needs to be culturally sensitive, taking into account traditional foods and social practices. In addition, nurses should offer individualized advice on safe fasting during Ramadan and address common misconceptions about insulin therapy. Sustained follow-up and effective communication are also essential for enhancing adherence and patient engagement.

At the health-system level, the findings point to the need for longer and more person-centered consultations, alongside strengthened follow-up—particularly through nurse-led or primary care–based services—that can offer ongoing, practical support. Such approaches may help bridge the gap between clinical recommendations and patients’ daily lives, making self-management more feasible and sustainable. In addition, the findings reinforce the value of proactive care, including pre-Ramadan assessment and individualized management planning, rather than reliance on generalized advice alone [41,50,51].

Collectively, the study themes reveal that diabetes self-management in Jordan and the region is not a knowledge-deficit issue but a cultural, social, emotional, and structural issue. Therefore, a remedial strategy is not only holistic but also cultural. In this regard, this study provides a social, cultural, and structural context for diabetes self-management. As such, it provides a foundation for a remedial strategy that is not only effective but also cultural and suitable for Jordan and the Arab region.

### Strengths and Limitations

This study has several strengths that merit mention. First, the depth to which the culturally grounded experiences of the participants were explored, which provided a rich source of insight into the ways in which social norms, religious beliefs, emotional pressures, and interactions with the healthcare system affect diabetes control. Second, the qualitative research design allowed participants to reveal the contradictions, dilemmas, and pressures that they faced, which might not be captured in a quantitative research design.

However, there are some limitations to the research. The participants were recruited based on their involvement in the healthcare system, which might mean that those who are not engaged in the system would be missed. Social desirability bias might also be a factor in the research, particularly in relation to culturally sensitive topics such as fasting or non-adherence to diet. In addition, although the interviews provided a cross-section of demographic backgrounds, the experiences of women who faced mobility issues could be further explored. Nevertheless, the similarities between the findings of the current study and the recent evidence‌‌ in the region suggest that the findings are relevant to the broader Middle Eastern community.

## Conclusion

This study highlights that diabetes self-management among adults in Jordan is shaped by a complex interplay of sociocultural norms, religious practices, emotional challenges, medication-related concerns, family expectations, and healthcare system constraints. These findings demonstrate that barriers to self-management are not merely individual issues, but are deeply embedded within patients’ lived contexts. Accordingly, effective diabetes care extends beyond individual knowledge and requires culturally sensitive, family-centered, and system-supported approaches.

Nurses play a key role in delivering culturally competent diabetes education that addresses traditional foods, family and social events, Ramadan fasting, medication adherence, and emotional well-being. Involving family members in care is essential, as they can significantly influence self-management practices.

From a policy and future practice perspective, diabetes care in Jordan should prioritize structured follow-up programs, nurse-led chronic disease management, family-centered education, tailored counseling for Ramadan fasting, and culturally appropriate physical activity initiatives. Interventions should also build on cultural and religious strengths by incorporating family support, faith-sensitive health messages, and practical guidance for managing diabetes during social and religious occasions.

## Supporting information

S1 Text Semi-structured interview guide for diabetes self-management. This file contains the semi-structured interview guide developed based on relevant literature and expert input. The guide was designed to explore participants’ lived experiences with type 2 diabetes self-management, including daily practices, perceived barriers, and facilitators related to diet, medication adherence, physical activity, and glucose monitoring. The open-ended questions allowed in-depth exploration and probing to ensure richness and credibility of the qualitative data.
(DOCX)
