# Supplementary material for: Lived experiences of type 2 diabetes self-management in Jordan: A qualitative study of sociocultural, religious, and healthcare influences
Source: PLoS One. 2026 Jun 5;21(6):e0350641. doi: 10.1371/journal.pone.0350641 (PMC13240902; doi:10.1371/journal.pone.0350641)
Supplement: S1 Text — This file contains the semi-structured interview guide developed based on relevant literature and expert input. The guide was designed to explore participants’ lived experiences with type 2 diabetes self-management, including daily practices, perceived barriers, and facilitators related to diet, medication adherence, physical activity, and glucose monitoring. The open-ended questions allowed in-depth exploration and probing to ensure richness and credibility of the qualitative data. (DOCX) [file pone.0350641.s001.docx]

| **The semi-structured interview guide** |
| --- |
| 1. Tell me about your experience in diabetes self-management. 2. Can you describe what managing diabetes looks like in your daily life? 3. What are the factors that you believe challenge your ability to manage your diet? 4. What factors influence your ability to follow your prescribed medications or insulin regimen? 5. What are the factors that you believe challenge your physical activity? 6. What are the factors that you believe challenge your ability to self-monitor glucose levels? 7. Overall, what influenced you to manage your diabetes by yourself? 8. In your opinion, what is the most important aspect of managing diabetes and why? 9. What are some facilitators that help you manage your diabetes by yourself? 10. What are some barriers to managing your diabetes by yourself? |
